# The Implicit Function as Squashing Time Model: A Novel Parallel Nonlinear EEG Analysis Technique Distinguishing Mild Cognitive Impairment and Alzheimer's Disease Subjects with High Degree of Accuracy

**DOI:** 10.1155/2007/35021

**Published:** 2007-11-25

**Authors:** Massimo Buscema, Massimiliano Capriotti, Francesca Bergami, Claudio Babiloni, Paolo Rossini, Enzo Grossi

**Affiliations:** ^1^Semeion Research Centre of Sciences of Communication, Via Sersale, 117, 00128 Rome, Italy; ^2^Department of Human Physiology and Pharmacology, University of Rome La Sapienza, 00185 Rome, Italy; ^3^Ospedale San Giovanni Calibita “Fatebenefratelli”, Isola Tiberina, 00153 Rome, Italy; ^4^Casa di cura San Raffaele Cassino (Frosinone), San Raffaele Pisana, Rome, Italy; ^5^IRCCS Centro San Giovanni di Dio Fatebenefratelli, 25100 Brescia, Italy; ^6^Department of Clinical Neurosciences, University of Rome Campus Biomedico, 00155 Rome, Italy; ^7^Bracco SpA Medical Department, Via E. Folli, 50, 20134 Milan, Italy

## Abstract

*Objective*. This paper presents the results obtained using a protocol based on special types of artificial neural networks (ANNs) assembled in a novel methodology able to compress the temporal sequence of electroencephalographic (EEG) data into spatial invariants for the automatic classification of mild cognitive impairment (MCI) and Alzheimer's disease (AD) subjects. With reference to the procedure reported in our previous study (2007), this protocol includes a new type of artificial organism, named TWIST. The working hypothesis was that compared to the results presented by the workgroup (2007); the new artificial organism TWIST could produce a better classification between AD and MCI. *Material and methods*. Resting eyes-closed EEG data were recorded in 180 AD patients and in 115 MCI subjects. The data inputs for the classification, instead of being the EEG data, were the weights of the connections within a nonlinear autoassociative ANN trained to generate the recorded data. The most relevant features were selected and coincidently the datasets were split in the two halves for the final binary classification (training and testing) performed by a supervised ANN. *Results*. The best results distinguishing between AD and MCI were equal to 94.10% and they are considerable better than the ones reported in our previous study (∼92%) (2007). *Conclusion*. The results confirm the working hypothesis that a correct automatic classification of MCI and AD subjects can be obtained by extracting spatial information content of the resting EEG voltage by ANNs and represent the basis for research aimed at integrating spatial and temporal information content of the EEG.

## 1. INTRODUCTION

The electroencephalogram (EEG), since its introduction, was considered the only methodology allowing a direct and online view of the “brain at work.” At the same time, abnormalities of the “natural” aging of the brain have yet been noticed in different types of dementias. The introduction of different structural imaging technologies in the 1970's and 1980's (computed tomography and magnetic resonance imaging) and the good results in the study of brain function obtained with techniques dealing with regional metabolism, glucose and oxygen consumption, and blood flow (single-photon emission computed tomography, positron emission tomography, functional magnetic resonance imaging) during the following two decades closet the role of EEG in a secondary line, particularly in the evaluation of Alzheimer's dementia (AD) and related dementias.

Lately, EEG computerized analysis in aged people has been enriched by various modern techniques able to manage the large amount of information on time-frequency processes at single recording channels (wavelet, neural networks, etc.) and on spatial localization of these processes [2–10]. The results have encouraged the scientific community in exploring electromagnetic brain activity, which changes by aging and can greatly deteriorate, through the different stages of the various forms of dementias. The use of neural networks represents an alternative and very promising attempt to make EEG analysis suitable for clinical applications in aging—thanks to their ability in extracting specific and smooth characteristics from huge amounts of data. Computerized processing of a large quantity of numerical data in wakeful relaxed subjects (“resting” EEG) made easier the automatic classification of the EEG signals, providing promising results even using relatively simple linear classifiers such as logistic regression and discriminant analysis. Using global field power (i.e., the sum of the EEG spectral power across all electrodes) as an input, some authors reached an accurate differential diagnosis between AD and MCI subjects with accuraces of 84% and 78%, respectively[11, 12]. Using evaluation of spectral coherence between electrode pairs (i.e., a measure of the functional coupling) as an input to the classification, the correct classification reached 82% when comparing the AD and normal aged subjects [13, 14].

Spatial smoothness and temporal fluctuation of the EEG voltage are considered as measures of the synaptic impairment, along with the notion that cortical atrophy can affect the spatiotemporal pattern of neural synchronization generating the scalp EEG. These parameters have been used to successfully discriminate the respective distribution of probable AD and normal aged subjects [15]. The interesting new idea in that study [15] was the analysis of resting EEG potential distribution instant by instant rather than the extraction of a global index along periods of tens of seconds or more.

Table 1 summarizes the results of a higher preclassification rate with ANN's analysis than with standard linear techniques, such as multivariate discriminatory analysis or the nearest-neighbour analysis [16]. Some authors [17] developed a system consisting of recurrent neural nets processing spectral data in the EEG. They succeeded in classifying AD patients and non-AD patients with a sensitivity of 80% and a specificity of 100%. In other studies, classifiers based on ANNs, wavelets, and blind source separation (BSS) achieved promising results [18, 19]. In a study from the same workgroup of this paper, we used a sophisticated technique based on blind source separation and wavelet preprocessing developed by Vialatte et al. [18] and Cichocki et al. [20–22] recently, whose results appear to be the best in the field when compared to the literature. We named this method *BWB model* (blind source separation + wavelet + bumping modeling), [1]. The results obtained in the classifications tasks, comparing AD patients to MCI subjects, using the BWB model, ranged from 78.85% to 80.43% (mean = 79.48%).

The aim of this study is to assess the strength of a novel parallel nonlinear EEG analysis technique in the differential classification of MCI subjects and AD patients, with a high degree of accuracy, based on special types of artificial neural networks (ANNs) assembled in a novel methodology able to compress the temporal sequence of electroencephalographic (EEG) data into spatial invariants. The working hypothesis is that this new approach to EEG based on nonlinear ANNs-based methods can contribute to improving the reliance of the diagnostic phase in association with other clinical and instrumental procedures. Compared to the results already presented by the workgroup [1], the included new artificial organism TWIST could produce a better classification between AD and MCI.

## 2. MATERIAL AND METHODS

The IFAST method includes two phases.

1. A squashing phase: an EEG track is compressed in order to project the *invariant patterns* of that track on the connections matrix of an autoassociated ANN. The EGG track/subject is now represented by a vector of weights, without any information about the target (AD or MCI).
2. “TWIST” (training with input selection and testing) phase: a technique of data resampling based on the genetic algorithm GenD, developed at Semeion Research Center. The new dataset which is composed by the connections matrix (output of the squashing phase), plus the target assigned to each vector, is splitted into two sub samples, each one for five times with a similar probability density function, in order to train, test, and validate the ANN models.

### 2.1. The IFAST method

#### 2.1.1. General philosophy

The core of this new methodology is that the ANNs do not classify subjects by directly using the EEG data as an input. Rather, the data inputs for the classification are the weights of the connections within a recirculation (nonsupervised) ANN trained to generate the recorded EEG data. These connection weights represent a model of the peculiar spatial features of the EEG patterns at the scalp surface. The classification, based on these weights, is performed by a standard supervised ANN.

This method, named IFAST (acronym for implicit function as squashing time), tries to understand the implicit function in a multivariate data series compressing the temporal sequence of data into spatial invariants and it is based on three general observations.

1. Every multivariate sequence of signals coming from the same natural source is a complex asynchronous dynamic highly nonlinear system, in which each channel's behavior is understandable only in relation to all the others.
2. Given a multivariate sequence of signals generated from the same source, the implicit function defining the above-mentioned asynchronous process is the conversion of that same process into a complex hypersurface, representing the interaction in time of all the channels' behavior.
3. The 19 channels in the EEG represent a dynamic system characterized by asynchronous parallelism. The nonlinear implicit function that defines them as a whole represents a metapattern that translates into space (hypersurface) that the interactions among all the channels create in time.

The idea underlying the IFAST method resides in thinking that each patient's 19-channel EEG track can be synthesized by the connection parameters of an autoassociated nonlinear ANN trained on the same track's data.

There can be several topologies and learning algorithms for such ANNs; what is necessary is that the selected ANN be of the autoassociated type (i.e., the input vector is the target for the output vector) and that the transfer functions defining it be*non linear and differentiable* at any point.

Furthermore, it is required that all the processing made on every patient be carried out with the same type of ANN, and that the initial randomly generated weights have to be *the same* in every learning trial. This means that, for every EEG, every ANN has to have the same starting point, even if that starting point is random.

We have operated in two ways in order to verify this method's efficiency.

1. Different experiments were implemented based on the same samples. By “experiment,” we mean a complete application of the whole procedure to every track of the sample.
2. The second way is using autoassociated ANNs with different topologies and algorithms on the entire sample in order to prove that any autoassociated ANN can carry out the task of *translating into the space domain* the whole EEG track through its connections.

#### 2.1.2. The squashing phase

The first application phase of the IFAST method may be defined as “*squashing*.” It consists in compressing an EEG track in order to project the *invariant patterns* of that track on the connections of an auto-associated ANN.

More formally

if

**Figure 1:**
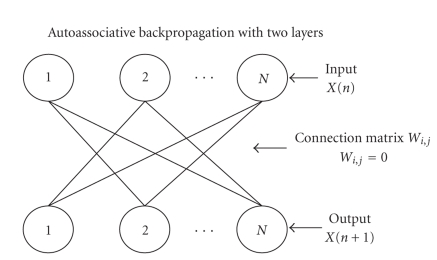
Autoassociative backpropagation ANN with $W_{j,j}=0$, as the connections on the main diagonal are not present.

$F_{i}\left(\right)=$ implicit function of the *i*-th EEG track
$X_{i}=$ matrix of the values of the *i*-th EEG
$W_{i_{j,k}}^{*}=$ trained matrix of the connections of the *i*-th EEG (* = objective of the squashing)
$W_{0_{j,k}}=$ random starting matrix, the same for all EEGs
  then in the case of a two-layered autoassociated ANN
$X_{i}=F_{i}\left(X_{i},W_{i_{j,k}}^{*},W_{0_{j,k}}\right);\;\text{con}\;W_{0_{j,j}}=0.$
$W_{i_{j,j}}=0$ means that every *i*th EEG track is processed by the two-layered autoassociated ANN in which $W_{j,j}=0$, as the connections on the main diagonal are not present (see Figure 1).

It is possible to use different types of autoassociated ANNs to run this search for spatial invariants in every EEG.

**Figure 4:**
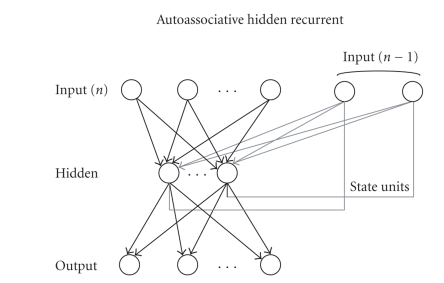
Elman's hidden recurrent ANN for auto-associating purposes using the backpropagation algorithm.

1. A backpropagation without a hidden unit layer and without connections on the main diagonal (for short, AutoBp):
  This is an ANN featuring an extremely simple learning algorithm:
  (1) $$\begin{array}{ll} {\text{Output}}_{i} & =f\left(\overset{N}{\underset{j}{\sum }}{\text{Input}}_{j}\cdot W_{i,j}+{\text{Bias}}_{i}\right)\\ & =\frac{1}{1+e^{-\left(\Sigma_{j}^{N}{\text{Input}}_{j}\cdot W_{i,j}+{\text{Bias}}_{i}\right)}},\;\;W_{i,i}=0;\\ \delta_{i} & =\left({\text{Input}}_{i}-{\text{Output}}_{i}\right)\cdot f^{\prime }\left({\text{Output}}_{i}\right)\\ & =\left({\text{Input}}_{i}-{\text{Output}}_{i}\right)\cdot {\text{Output}}_{i}\cdot (1-{\text{Output}}_{i});\\ \Delta W_{i,j} & =\text{LCoef}\cdot \delta_{i}\cdot {\text{Input}}_{j},\;\;\;\;\text{LCoef}\in \left[\text{0,1}\right],\\ \Delta {\text{Bias}}_{i} & =\text{LCoef}\cdot \delta_{i}. \end{array}$$
  AutoBP is an ANN featuring $N^{2}-N$ internode connections and N bias inside every exit node, for a total of $N^{2}$ adaptive weights. This algorithm works similarly to logistic regression and can be used to establish the dependency of variables from each others.
  The advantage of AutoBP is due to its learning speed, in turn due to the simplicity of its topology and algorithm. Moreover, at the end of the learning phase, the connections between variables, being direct, have a clear conceptual meaning. Every connection indicates a relationship of faded excitement, inhibition, or indifference between every pair of channels in the EEG track of any patient.
  The disadvantage of AutoBP is its limited convergence capacity, due to that same topological simplicity. That is to say, complex relationships between variables may be approximated or ignored (for details, see [23, 24]).
2. New recirculation network (for short, NRC) is an original variation [25] of an ANN that has existed in the literature [26] and was not considered to be useful to the issue of autoassociating between variables.
  The topology of the NRC which we designed includes only one connection matrix and four layers of nodes: one input layer, corresponding to the number of variables; one output layer whose target is the input vector; two layers of hidden nodes with the same cardinality independent from the cardinality of the input and output layers. The matrix between input-output nodes and hidden nodes is fully connected and in every learning cycle, it is modified both ways, according to the following equations:
  (2) $$\begin{array}{ll} {\text{Hidden1}}_{i} & =f\left(\overset{N}{\underset{j}{\sum }}{\text{Input}}_{j}\cdot W_{i,j}+{\text{BiasHidden}}_{i}\right)\\ & =f\left({\text{Net}}_{i}^{\text{Hidden1}}\right)=\frac{1}{1+e^{-{\text{Net}}_{i}^{H1}}};\\ {\text{Output}}_{j} & =R\cdot {\text{Input}}_{j}+\left(1-R\right)\\ & \;\cdot f\left(\overset{M}{\underset{i}{\sum }}{\text{Hidden1}}_{i}\cdot W_{j,i}+{\text{BiasOutput}}_{j}\right)\\ & =R\cdot {\text{Input}}_{j}+\left(1-R\right)\cdot f\left({\text{Net}}_{j}^{\text{Output}}\right)\\ & =R\cdot {\text{Input}}_{j}+\left(1-R\right)\cdot \frac{1}{1+e^{-{\text{Net}}_{j}^{\text{Output}}}};\\ & R\in \left[0,1\right]/{}^{*}\text{Projection}\;\text{Coefficient}{}^{*}/\\ \text{Hidden}2_{i} & =R\cdot \text{Hidden}1_{i}+\left(1-R\right)\\ & \;\cdot f\left(\overset{N}{\underset{j}{\sum }}{\text{Output}}_{j}\cdot W_{i,j}+{\text{BiasHidden}}_{i}\right)\\ & =R\cdot \text{Hidden}1_{i}+\left(1-R\right)\cdot f\left({\text{Net}}_{i}^{\text{Hidden}2}\right)\\ & =R\cdot \text{Hidden}2_{i}+\left(1-R\right)\cdot \frac{1}{1+e^{-{\text{Net}}_{i}^{\text{Hidden}2}}};\\ \Delta W_{j,i} & =\text{LCoef}\cdot \left({\text{Input}}_{j}-{\text{Output}}_{j}\right)\cdot \text{Hidden}1_{i};\\ \Delta {\text{BiasOutput}}_{j} & =\text{LCoef}\cdot \left({\text{Input}}_{j}-{\text{Output}}_{j}\right);\\ & \text{LCoef}\in \left[0,1\right]/{}^{*}\text{Learning}\;\text{Coefficient}{}^{*}/\\ \Delta W_{i\cdot i} & =\text{LCoef}\cdot \left(\text{Hidden}1_{i}-\text{Hidden}2_{i}\right)\cdot {\text{Output}}_{j};\\ \Delta \text{BiasHidden}{}_{i} & =\text{LCoef}\cdot \left(\text{Hidden}1_{i}-\text{Hidden}2_{i}\right). \end{array}$$
  NRC then features $N^{2}$ internode adaptive connections and 2⋅N intranode adaptive connections (bias). The advantages of NRC are its excellent convergence ability on complex datasets and, as a result, an excellent ability to interpolate complex relations between variables.
  The disadvantages mainly have to do with the vector codification that the hidden units run on the input vectors making the conceptual decoding of its trained connections difficult.
3. Autoassociative multilayer perceptron (for short, AMLP) may be used with an auto-associative purpose (encoding)— thanks to its hidden units layer, that decomposes the input vector into main nonlinear components. The algorithm used to train the MLP is a typical backpropagation algorithm [27].
  The MLP, with only one layer of hidden units, features two connection matrices and two intranode connection vectors (bias), according to the following definitions:
*N* = number of input variables = number of output variables;
*M* = number of nodes in the hidden layer;
*C* = total number of internode and intranode connections (bias);
    (3) C=2⋅N⋅M+N+M.
    The advantages of MLP are its well-known flexibility and the strength of its backpropagation algorithm. Its disadvantages are the tendency to saturate the hidden nodes in the presence of nonstationary functions, and the vector codification (allocated) of the same hidden nodes.
4. Elman's hidden recurrent [28] can be used for autoassociating purposes, again using the backpropagation algorithm (for short, autoassociative hidden recurrent AHR, see Figure 4). It was used in our experimentation as a variation for MLP with memory set to one step. It is not possible to call it a proper recurring ANN in this form, because the memory would have been limited to one record before. We used this variation only to give the ANN an input vector modulated at any cycle by the values of the previous input vector. Our purpose was not to codify the temporal dependence of the entrance signals, but rather to give the ANN a “smoother” and more mediated input sequence. The number of connections in the AHR BP is the same as an MLP with extended input, whose cardinality is equal to the number of hidden units:
  (4) $$C=2\cdot N\cdot M+N+M+M^{2}.$$

The software IFAST (developed in Borland *C*) [29] produces the squashing phase through the training operated by these four networks; in the “MetaTask” section the user can define the whole procedure by selecting

- the files that will be processed (in our case every complete EEG),
- the type of network,
- the sequence of the records for every file (generally random),
- the number of epochs of training,
- a training stop criterion (number of epochs or minimum RMSE),
- the number of hidden nodes of the autoassociated network, which determines the length of the output vector of the file processed
- the number of matrices, depending on the type of the autoassociated network selected,
- the learning coefficient and delta rate.

### 2.2. TWIST

From this phase, the procedure is completely different from the one described in our precedent work [1]. The choice of following a different methodology was due to the will of improving the classification results and removing causes of loss of information.

In the former study, the dataset coming from the squashing phase was compressed by another autoassociated ANN, in the attempt of eliminating the invariant pattern, codified from the previous ANN, relating to specific characteristic of the brain (anxiety level, background level, etc.) which is not useful for the classification, leaving the most significant ones unaltered. Then the new compressed datasets were split into two halves, (training and test) using T&T [30] evolutionary algorithm, for the final binary classification.

Rather in this work, the elimination of the noisiest features and the classification run parallel to each other. We will show that the new procedure has obtained better performances.

First of all, a new dataset called “Diagnostic DB” was created for easier understanding. The diagnostic gold standard has been established, for every patient, in a way that is completely independent of the clinical and instrumental examinations (magnetic resonance imaging, etc.) carried out by a group of experts whose diagnosis has been also reconfirmed in time.

The diagnoses have been divided into the following two classes, based on delineated inclusion criteria:

- elderly patients with “cognitive decline” (MCI);
- elderly patients with “probable Alzheimer” (AD);

We rewrote the last generated dataset, adding to every $H_{n_{s}}$ vector the diagnostic class that an objective clinical examination had assigned to every patient. The $H_{m_{s}}$ vectors represent the invariant traits *s* as defined by the squashing phase for every *m*-th subject EEG track, that is, the columns number of the connections matrix depending on the specific autoassociated network used.

Then the dataset is ready for the next step. This new phase is called TWIST [31] and includes the utilization of two systems T&T and IS [30], both based on a genetic algorithm, GenD, developed at Semeion Research Centre [32].

T&T systems are robust data resampling techniques able to arrange the source sample into subsamples, each one with a similar probability density function. In this way the data split into two or more subsamples in order to train, test, and validate the ANN models more effectively.

The IS system is an evolutionary system for feature selection based on a wrapper approach. While the filter approach looks at the inner properties of a dataset providing a selection that is independent of the classification algorithm to be used afterwards, in the wrapper approach various subsets of features are generated and evaluated using a specific classification model using its performances as a guidance to optimization of subsets.

The IS system reduces the amount of data while conserving the largest amount of information available in the dataset. The combined action of these two systems allows us to solve two frequent problems in managing artificial neural networks:

1. the size and quality of the training and testing sets,
2. the large number of variables which, apparently, seem to provide the largest possible amount of information. Some of the attributes may contain redundant information, which is included in other variables, or confused information (noise) or may not even contain any significant information at all and be completely irrelevant.

Genetic algorithms have been shown to be very effective as global search strategies when dealing with nonlinear and large problems.

The “*training and testing*” algorithm (T&T) is based on a population of *n* ANNs managed by an evolutionary system. In its simplest form, this algorithm reproduces several distribution models of the complete dataset *D_*Γ*_* (one for every ANN of the population) in two subsets ($d_{\Gamma }^{\left[\text{t}\text{r}\right]}$, the training set, and $d_{\Gamma }^{\left[\text{t}\text{s}\right]}$, the testing set). During the learning process each ANN, according to its own data distribution model, is trained on the subsample $d_{\Gamma }^{\left[\text{t}\text{r}\right]}$ and blind-validated on the subsample $d_{\Gamma }^{\left[\text{t}\text{s}\right]}$.

The performance score reached by each ANN in the testing phase represents its “fitness” value (i.e., the individual probability of evolution). The genome of each “network individual” thus codifies a data distribution model with an associated validation strategy. The *n* data distribution models are combined according to their fitness criteria using an evolutionary algorithm. The selection of “network individuals” based on fitness determines the evolution of the population, that is, the progressive improvement of performance of each network until the optimal performance is reached, which is equivalent to the better division of the global dataset into subsets. The evolutionary algorithm mastering this process, named “genetic doping algorithm” (GenD for short), created at Semeion Research Centre, has similar characteristics to a genetic algorithm [33–37] but it is able to maintain an inner instability during the evolution, carrying out a natural increase of biodiversity and a continuous “evolution of the evolution” in the population.

The elaboration of T&T is articulated in two phases.

In a preliminary phase, an evaluation of the parameters of the fitness function that will be used on the global dataset is performed. The configuration of a standard backpropagation network that most “suits” the available dataset is determined: the number of layers and hidden units, some possible generalizations of the standard learning law, the fitness values of the population's individuals during evolution. The parameters thus determined define the configuration and the initialization of all the individual networks of the population and will then stay fixed in the following computational phase. The accuracy of the ANN performance with the testing set will be the fitness of that individual (i.e., of that hypothesis of distribution into two halves of the whole dataset).

In the computational phase, the system extracts from the global dataset the best training and testing sets. During this phase, the individual network of the population is running, according to the established configuration and the initialization parameters.

Parallel to T&T runs “*Input Selection*” (IS), an adaptive system, based on the same evolutionary algorithm GenD, consisting of a population of ANN, in which each one carries out a selection of the independent and relevant variables on the available database.

The elaboration of IS, as for T&T, is developed in two phases. In the preliminary phase, a standard backpropagation ANN is configured in order to avoid possible over fitting problems. In the computational phase, each individual network of the population, identified by the most relevant variables, is trained on the training set and tested on the testing set.

The evolution of the individual network of the population is based on the algorithm GenD. In the I.S. approach, the GenD genome is built by *n* binary values, where *n* is the cardinality of the original input space. Every gene indicates if an input variable is to be used or not during the evaluation of the population fitness. Through the evolutionary algorithm GenD, the different “hypotheses” of variable selection, generated by each ANN of the population, change over time, at each generation; this leads to the selection of the best combination of input variables. As in the T&T systems, the genetic operators crossover and mutation are applied on the ANNs population; the rates of occurrence for both operators are self-determined by the system in an adaptive way at each generation.

When the evolutionary algorithm no longer improves its performance, the process stops, and the best selection of the input variables is employed on the testing subset.

The software based on TWIST phase algorithm (developed in *C*-Builder [31]) allows the configuration of the genetic algorithm GenD:

- the population (the number of individual networks),
- number of hidden nodes of the standard BP,
- number of epochs,
- the output function SoftMax,
- the cost function (classification rate in our case).

The generated outputs are the couple of files SetA and SetB (subsets of the initial db defined by the variables selected) that will be used in the validation protocol (see Section 2.3).

### 2.3. The validation protocol

**Figure 6:**
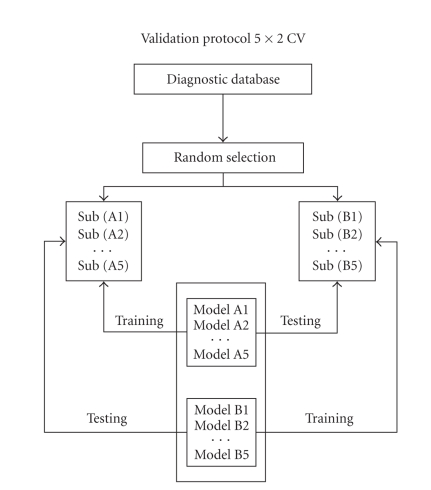
5 × 2 validation protocol for the independent identification of the spatial invariants of EEGs.

The validation protocol is a fundamental procedure to verify the models' ability to generalize the results reached in the Testing phase of each model. The application of a fixed protocol measures the level of performance that a model can produce on data that are not present in the testing and/or training sample. We employed the so-called 5 × 2 cross-validation protocol (see Figure 6) [38]. This is a robust protocol that allows one to evaluate the allocation of classification errors. In this procedure, the study sample is randomly divided ten times into two subsamples, always different but containing a similar distribution of cases and controls.

The ANNs' good or excellent ability to diagnostically classify all patients in the sample from the results of the confusion matrices of these 10 independent experiments would indicate that the spatial invariants extracted and selected with our method truly relate to the functioning quality of the brains examined through their EEG.

### 2.4. Experimental setting

#### 2.4.1. Subjects and diagnostic criteria

The population study included

- 180 AD patients (gender: 50 males/130 females; age: mean = 77 ± 6.78 SD, range from 54 to 91; MMSE: mean = 19.9, ± 4.89 SD, range from 5 to 30);
- 115 MCI subjects (gender: 49 males/66 females; age: mean = 76 ± 6.37 SD, range from 42 to 88; MMSE: mean = 25.2, ± 2.35 SD, range from 17.3 to 29).

The samples were matched for age, gender, and years of education. Part of the individual data sets was used for previous EEG studies [2–4]. In none of these studies we addressed the specific issue of the present study. Local institutional ethics committees approved the study. All experiments were performed with the informed and overt consent of each participant or caregiver.

The present inclusion and exclusion criteria for MCI were based on previous seminal studies [39–46] and designed for selecting elderly persons manifesting objective cognitive deficits, especially in the memory domain, who did not meet criteria for a diagnosis of dementia or AD, namely, with, (i) objective memory impairment on neuropsychological evaluation, as defined by performances ≥1.5 standard deviation below the mean value of age and education-matched controls for a test battery including memory rey list (immediate recall and delayed recall), Digit forward and Corsi forward tests; (ii) normal activities of daily living as documented by the patient's history and evidence of independent living; (iii) clinical dementia rating score of 0.5; (iv) geriatric depression scale scores < 13.

Exclusion criteria for MCI were: (i) mild AD, as diagnosed by the procedures described above; (ii) evidence of concomitant dementia such as frontotemporal, vascular dementia, reversible dementias (including pseudodepressive dementia), fluctuations in cognitive performance, and/or features of mixed dementias; (iii) evidence of concomitant extrapyramidal symptoms; (iv) clinical and indirect evidence of depression lower than 14 as revealed by GDS scores; (v) other psychiatric diseases, epilepsy, drug addiction, alcohol dependence, and use of psychoactive drugs including acetylcholinesterase inhibitors or other drugs enhancing brain cognitive functions; (vi) current or previous systemic diseases (including diabetes mellitus) or traumatic brain injuries.

Probable AD was diagnosed according to NINCDS-ADRDA criteria [47]. Patients underwent general medical, neurological, and psychiatric assessments and were also rated with a number of standardized diagnostic and severity instruments that included MMSE [48], clinical dementia rating scale [49], geriatric depression scale [50], Hachinski ischemic scale [51], and instrumental activities of daily living scale [52]. Neuroimaging diagnostic procedures (computed tomography or magnetic resonance imaging) and complete laboratory analyses were carried out to exclude other causes of progressive or reversible dementias, in order to have a homogenous probable AD patient sample. The exclusion criteria included, in particular, any evidence of (i) front temporal dementia diagnosed according to criteria of Lund and Manchester groups [53]; (ii) vascular dementia as diagnosed according to NINDS-AIREN criteria [54] and neuroimaging evaluation scores [55, 56]; (iii) extra pyramidal syndromes; (iv) reversible dementias (including pseudo dementia of depression); (v) Lewy body dementia according to the criteria by McKeith et al. [57]. It is important to note that benzodiazepines, antidepressant, and/or antihypertensive drugs were withdrawn for about 24 hours before the EEG recordings.

#### 2.4.2. EEG recordings

EEG data were recorded in wake rest state (eyes-closed), usually during late morning hours from 19 electrodes positioned according to the international 10–20 system (i.e., Fp1, Fp2, F7, F3, Fz, F4, F8, T3, C3, Cz, C4, T4, T5, P3, Pz, P4, T6, O1, O2; 0.3–70 Hz filtering band passes). A specific reference electrode was not imposed to all recording units of this multi-centric study, since any further data analysis was carried out after EEG data were rereferenced to a common average reference. The horizontal and vertical electrooculogram was simultaneously recorded to monitor eye movements. An operator controlled, online, the subject and the EEG traces by alerting the subject any time there were signs of behavioural and/or EEG drowsiness in order to keep the level of vigilance constant. All data were digitized (5 minutes of EEG; 0.3–35 Hz band pass 128 Hz sampling rate).

The duration of the EEG recording (5 minutes) allowed the comparison of the present results with several previous AD studies using either EEG recording periods shorter than 5 minutes [58–62] or shorter than 1 minute [7, 8]. Longer resting EEG recordings in AD patients would have reduced data variability, but they would have increased the possibility of EEG “slowing” because of reduced vigilance and arousal.

EEG epochs with ocular, muscular, and other types of artefact were preliminarily identified by a computerized automatic procedure. Those manifesting sporadic blinking artefacts (less than 15% of the total) were corrected by an autoregressive method [63].

The performances of the software package on EOG-EEG-EMG data related to cognitive-motor tasks were evaluated with respect to the preliminary data analysis performed by two expert electroencephalographists (gold standard). Due to its extreme importance for multicentric EEG studies, we compared the performances of two representative “regression” methods for the EOG correction in time and frequency domains. The aim was the selection of the most suitable method in the perspective of a multicentric EEG study. The results showed an acceptable agreement of approximately 95% between the human and software behaviors, for the detection of vertical and horizontal EOG artifacts, the measurement of hand EMG responses for a cognitive-motor paradigm, the detection of involuntary mirror movements, and the detection of EEG artifacts. Furthermore, our results indicated a particular reliability of a “regression” EOG correction method operating in time domain (i.e., ordinary least squares). These results suggested the use of the software package for multicentric EEG studies.

Two independent experimenters—blind to the diagnosis—manually confirmed the EEG segments accepted for further analysis. A continuous segment of artefact-free EEG data lasting for 60 seconds was used for subsequent analyses for each subject.

#### 2.4.3. Preprocessing protocol

The entire sample of 466 subjects was recorded at 128 Hz for 1 minute. The EEG track of each subject was represented by a matrix of 7680 sequential rows (time) and 19 columns (the 19 channels).

The *squashing phase* was implemented using the four autoassociative ANNs described [29]:

- an autoassociative BP with 2 layers (ABP);
- a new recirculation ANN (NRC);
- an autoassociative multilayer perceptron with 3 layers (AMLP);
- an autoassociative hidden recurrent (AHR).

Every autoassociative ANN independently processed every EEG of the total sample in order to assess the different capabilities of each ANN to extract the key information from the EEG tracks.

After this processing, each EEG track is squashed into the weights of every ANN resulting in 4 different and independent datasets (one for each ANN), whose records are the squashing of the original EEG tracks and whose variables are the trained weights of every ANN.

After TWIST processing, the most significant features for the classification were selected and at the same time the training set and the testing set with a similar function of probability distribution that provides the best results in the classification were defined.

The validation protocol 5x2CV was applied blindly to test the capabilities of a generic supervised ANN to correctly classify each record (the number of inputs depending on the number of variables selected by IS).

A supervised MLP was used for the classification task, without hidden units. In every experimentation, in fact, we were able to train perfectly the ANN in no more than 100 epochs (root mean square error (RMSE) < 0.0001). That means that in this last phase, we could have used also a linear classifier to reach up the same results.

## 3. RESULTS

The experimental design consisted in 10 different and independent processing for the classification AD versus MCI. Every experiment was conducted in a blind and independent manner in two directions: training with subsample A and blind testing with subsample B versus training with subsample B and blind testing with subsample A.

Table 3 shows the mean results summary for the classifications of AD versus MCI, compared to the results obtained in the experimentations reported in a previous study [1], based on a different protocol (without the TWIST phase).

Regarding the protocol IFAST-TWIST, the ABP and AHR achieved the best results comparing AD with MCI subjects (94.10% and 93.36%), but all the performances are considerably better than those obtained in the previous study.

Tables 4, 5, 6 and 7 show the details of the results obtained by each autoassociated ANN, where

**Figure 8:**
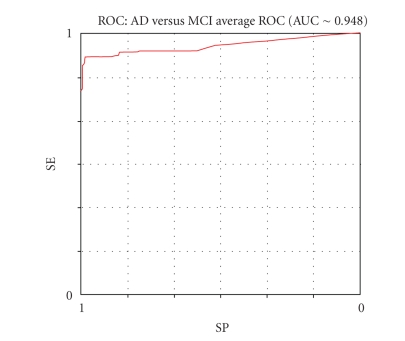
The average ROC curve of the ABP performance (threshold method).

**Figure 9:**
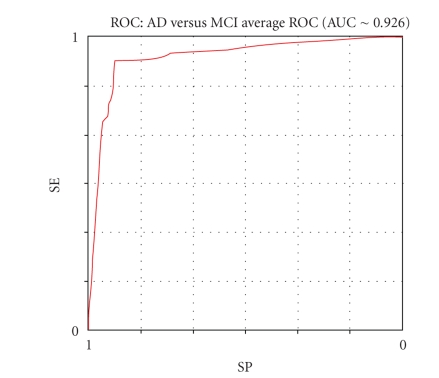
The average ROC curve of the NRC performance (threshold method).

**Figure 10:**
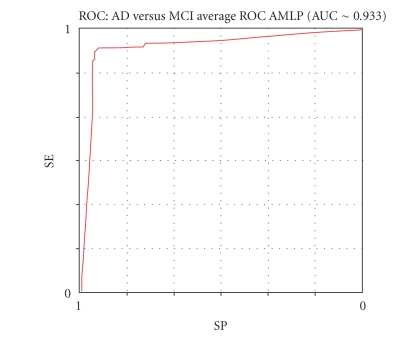
The average ROC curve of the AMLP performance (threshold method).

**Figure 11:**
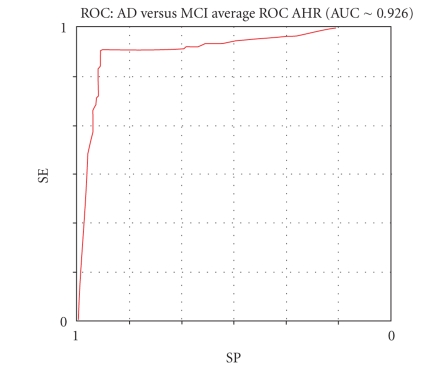
The average ROC curve of the AHR performance (threshold method).

SE = sensibility,
SP = specificity,
VP+ = positive predictive value,
VP− =negative predictive value,
LR+ = likelihood ratio for positive test results (benchmark value ≥ 2),
LR− = likelihood ratio for negative test results (benchmark value ≤ 0.2),
AUC = area under ROC curve (average ROC curve calculated by the threshold method),

Figures 8, 9, 10, and 11 show the respective average Roc curves.

## 4. DISCUSSION

Various types of nonreversible forms of dementias represent a major health problem in all those countries where the average life span is progressively increasing. There is a growing amount of scientific and clinical evidences that brain neural networks rearrange their connections and synapses to compensate neural loss due to neuro degeneration [64]. This process of plasticity maintains brain functions at an acceptable level before clear symptoms of dementia appear. The length of this presymptomatic period is currently unknown but, in the case of AD, often preceded by MCI, it lasts several years. Despite the lack of an effective treatment, able to block progression and/or to reverse the cognitive decline, it is generally agreed that early beginning of the available treatment (i.e., inhibitors of anticholinesterase drugs) provides the best results [65]. A significant advancement in the fight against dementias would be to have in our hands a non-invasive, easy-to-perform, and low-cost diagnostic tool capable of screening with a high rate of positive prognostication a large at-risk population sample (i.e., MCI, subjects with genetic defects and a family history of dementias or other risk factors). To test this issue, we performed automatic classification of MCI and AD subjects extracting with ANNs the spatial content of the EEG voltage. The results showed that the correct automatic classification rate reached 94.10% for AD versus MCI, better than the classification rate obtained with the more advanced currently available nonlinear techniques. These results confirm the working hypothesis that this EEG approach based on ANNs can contribute to improve the precision of the diagnostic phase in association with other clinical and instrumental procedures.

The present results suggest that the present variant of IFAST procedure (TWIST) could be used for a large screening of MCI subjects under control, to detect the first signs of conversion to AD for triggering further clinical and instrumental evaluations crucial for an early diagnosis of AD (this is invaluable for the beginning of cholinergic therapies that are generally carried out only in overt AD patients due to gastro intestinal side effects). Indeed, the actual percentage of correct discrimination between MCI and probable AD is around 94%. This rate is clearly insufficient for the use of the IFAST procedure for a diagnosis, due to 6% of misclassifications. The present results prompt future studies on the predictive value of cortical EEG rhythms in the early discrimination of MCI subjects who will convert to AD. This interesting issue could be addressed by a proper longitudinal study. MCI subjects should be divided into “converted” and “stable” subgroups, according to final out-come as revealed by followup after about 5 years (i.e., the period needed for conversion of all MCI subjects fated to decline over time based on the mentioned literature). That study should demonstrate that the spatial EEG features at baseline measurement as revealed by the IFAST procedure might be discriminated between MCI converted and MCI stable subjects. Furthermore, baseline values of spatial EEG features in individual MCI subjects should be successfully used as an input by the IFAST procedure to predict the conversion to dementia. This intriguing research perspectives are the sign of the heuristic value of the present findings. However, apart from clinical perspectives, the present findings have an intrinsic value for clinical neurophysiology. They provided further functional data from a large aged population to support the idea that spatial features of EEG, as a reflection of the cortical neural synchronization, convey information content able to discriminate preclinical stage of dementia (MCI) from probable AD.

Furthermore, the evaluation of that diagnostic contribution may motivate future scientific studies probing its usefulness for prognosis and monitoring of AD across temporal domain.

Although EEG would fulfil up all the previous requirements, the way in which it is currently utilized does not guarantee its ability in the differential diagnosis of MCI, early AD, and healthy nonimpaired aged brains. The neurophysiologic community always had the perception that there is much more information about brain functioning embedded in the EEG signals than those actually extracted in a routine clinical context. The obvious consideration is that the generating sources of EEG signals (cortical postsynaptic currents at dendritic tree level) are the same ones as those attacked by the factors producing symptoms of dementia. The main problem is that usually in the signal-to-noise ratio the latter is largely overcoming the former.

This paper suggests that the reasons why the clinical use of EEG has been somewhat limited and disappointing with respect to early diagnosis of AD and identification of MCI—despite the progresses obtained in recent years—are due to the following, erring, general principles:

- identify and synthesizing the mathematical components of the signal coming from each *individual* recording site, considering the EEG channel as exploring only one, discrete brain area under the exploring electrode, and suming up all of them in attempt to reconstruct the general information;
- focusing on the time variations of the signal coming from each *individual* recording site,
- mainly employing linear analysis instruments.

The basic principle which is proposed in this work is very simple; *all* the signals from *all* the recording channels are analyzed together—and not individually—in both time and space. The reason for such an approach is quite simple; the instant value of the EEG in any recording channel depends, in fact, upon its previous and following values, and upon the previous and following values of *all* the other recording channels.

We believe that the EEG of each individual subject is defined by a specific *background signal model*, distributed in time and in the space of the recording channels (19 in our case). Such a model is a set of background invariant features able to specify the quality (i.e., cognitive level) of the brain activity, even in so a called resting condition. We all know that the brain never rests, even with closed eyes and if the subject is required to relax. The method that we have applied in this research context completely ignores the subject's contingent characteristics (age, cognitive status, emotions, etc.). It utilized a recurrent procedure which *squeezes* the significant signal and progressively selects the features useful for the classification.

## 5. CONCLUSIONS

We have tested the hypothesis that a correct automatic classification of MCI and AD subjects can be obtained extracting spatial information content of the resting EEG voltage by ANNs. The spatial content of the EEG voltage was extracted by a novel step-wise procedure. The core of this procedure was that the ANNs did not classify individuals using EEG data as an input; rather, the data inputs for the classification were the weights of the connections within an ANN trained to generate the recorded EEG data. These connection weights represented a useful model of the peculiar spatial features of the EEG patterns at scalp surface. Then the new system TWIST, based on a genetic algorithm, processed the weights to select the most relevant features and at the same time to create the best subset, training set, and testing set, for the classification. The results showed that the correct automatic classification rate reached 94.10% for AD versus MCI. The results obtained are superior to those obtained with the more advanced currently available nonlinear techniques. These results confirm the working hypothesis and represent the basis for research designed to integrate EEG-derived spatial and temporal information content using ANNs.

From methodological point of view, this research shows the need to analyze the 19 EEG channels of each person as a whole complex system, whose decomposition and/or linearization can involve the loss of many key information.

The present approach extends those of previous EEG studies applying advanced techniques (wavelet, neural networks, etc.) on the data of single recording channels; it also complements those of previous EEG studies in aged people, evaluating the spatial distributions of the EEG data instant by instant and the brain sources of these distributions [2–10].

With complex systems, it is not possible to establish a priori which information is relevant and which is not. Nonlinear autoassociative ANNs are a group of methods to extract from these systems the maximum of linear and nonlinear associations (features) able to explain their “strange” dynamics.

This research also documents the need to use different architectures and topologies of ANNs and evolutionary systems within complex procedures in order to optimize a specific medical target. This study's EEG analysis used

1. different types of nonlinear autoassociative ANNs for squashing data;
2. a new system, TWIST, based on a genetic algorithm, which manages supervised ANNs in order to select the most relevant features and to optimize the distribution of the data in training and testing sets;
3. a set of supervised ANNs for the final patterns recognition task.

It is reasonable to conclude that ANNs and other adaptive systems should be used as cooperative adaptive agents within a structured project for complex, useful applications.

## Figures and Tables

**Figure 2:**
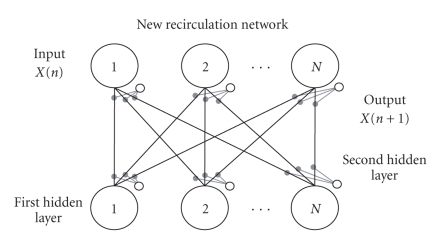
New recirculation network (NRC), with one connection matrix and four layers of nodes: one input layer, one output layer, and two layers of hidden nodes.

**Figure 3:**
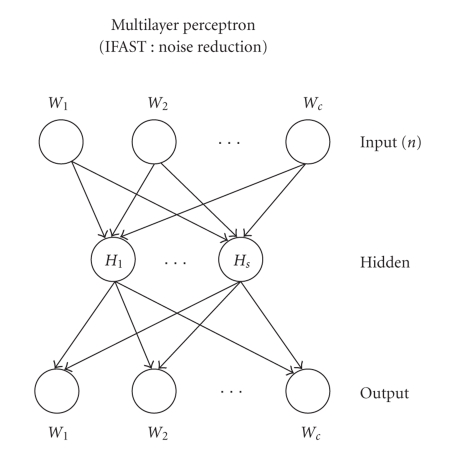
Multilayer perceptron; its hidden units layer decomposes the input vector into main nonlinear components.

**Figure 5:**
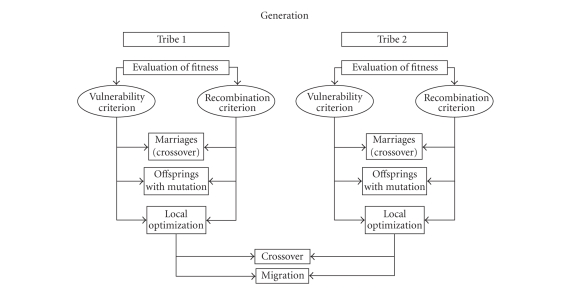
The structure and the operators of the evolutionary algorithm GenD.

**Figure 7:**
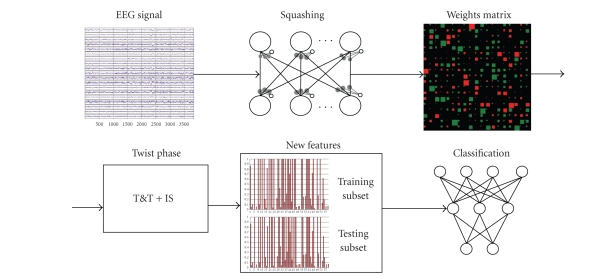
Procedure's scheme: from the squashing phase applied to EEG signal, the TWIST phase, to the final classification phase by ANNs.

**Table 1:** EEG automatic classification (* = severe AD ** = mild AD; S. no. = Sample; N. aged = normal aged; ANN = artificial neural networks; LDA = linear discriminant analysis; ACC = accuracy (%); SE = sensibility; SP = specificity).

Author year	S. no.	AD	N. aged	MCI	Length (s)	Classificators		ACC	SE	SP
						ANN	LDA			
Pritchard et al. (1994)	39	14	25		nd	x	x	85	nd	nd
Besthorn et al. (1997)	nd	nd	nd		nd	x	x	86.60		
Huang et al. [6, 11]	93	38	24	31	nd		x	81	84	78
Knott et al. (2001)	65	35	30		nd		x	75		
Petrosian et al. [17]	20	10	10		120	x		90	80	100
Cichocki et al. [20]	60		38	22	20		x	78.25	73	84
Melissant et al. [16]	36	15*	21		40	x		94	93	95
Melissant et al. [16]	38	28**	10		40	x		82	64	100

**Table 2:** Autoassociative ANN types and parameters used during the processing.

ANN parameters type	AbP	NRC	AMLP	AHR
Number of inputs	19	19	19	19
Number of outputs	19	19	19	19
Number of state units	0	0	0	10
Number of hidden units	0	19	10	10
Number of weights	361	399	409	509
Number of epochs	200	200	200	200
Learning coefficient	0.1	0.1	0.1	0.1
Projection coefficient	Null	0.5	Null	Null

**Table 3:** Summary and comparison of AD results versus MCI.

Blind classification	AD versus MCI		
Type of input vector	Sensitivity	Specificity	Accuracy
ABP	90.73	97.46	94.1
NRC	89.27	93.32	91.29
AMLP	92.42	94.14	93.28
AHR	92.11	92.61	92.36

**Table 4:** Details of the ABP results.

ABP results (%)										
ANN	SE	SP	A.MeanAcc.	W.MeanAcc.	Errors	VP+	VP−	LR+	LR−	AUC
FF_Bp(ab)	97.14	94.92	96.03	96.12	5	95.77	96.55	19.1	0.03	∼ 0.98
FF_Bp(ba)	84.31	100	92.16	89.87	16	100	77.78	+ Inf	0.16	∼ 0.928

Mean results	90.73	97.46	94.1	93	10.5	97.88	87.17	+ Inf	0.1	∼ 0.948

*Average ROC curve calculated by the threshold method.

**Table 5:** Details of the NRC results.

NRC results (%)										
ANN	SE	SP	A.MeanAcc.	W.MeanAcc.	Errors	VP+	VP−	LR+	LR−	AUC
FF_Bp(ab)	84.16	96.15	90.16	88.24	18	97.7	75.76	21.88	0.16	∼ 0.898
FF_Bp(ba)	94.37	90.48	92.42	92.54	10	91.78	93.44	9.91	0.06	∼ 0.932

Mean results	89.27	93.32	91.29	90.39	14	94.74	84.6	15.90	0.11	∼ 0.926

**Table 6:** Details of the AMLP results.

AMLP results (%)										
ANN	SE	SP	A.MeanAcc.	W.MeanAcc.	Errors	VP+	VP−	LR+	LR−	AUC
FF_Bp(ab)	93.26	92.19	92.72	92.81	6	94.32	90.77	11.94	0.07	∼ 0.930
FF_Bp(ba)	91.57	96.08	93.82	93.28	7	97.44	87.5	23.35	0.09	∼ 0.935

Mean results	92.42	94.14	93.28	93.05	6.5	95.88	89.14	17.65	0.08	∼ .933

**Table 7:** Details of the AHR results.

AHR results (%)										
ANN	SE	SP	A.MeanAcc.	W.MeanAcc.	Errors	VP+	VP−	LR+	LR−	AUC
FF_Bp(ab)	97.22	89.23	93.23	93.43	9	90.91	96.67	9.03	0.03	∼ 0.940
FF_Bp(ba)	87	96	91.5	90	15	97.75	78.69	21.75	0.14	∼ 0.904

Mean results	92.11	92.62	92.37	91.72	12	94.33	87.68	15.39	0.09	∼ 0.926

## References

[1] Buscema M, Rossini P, Babiloni C, Grossi E (2007). The IFAST model, a novel parallel nonlinear EEG analysis technique, distinguishes mild cognitive impairment and Alzheimer's disease patients with high degree of accuracy. *Arificial Intelligence in Medicine*.

[2] Babiloni C, Binetti G, Cassetta E (2004). Mapping distributed sources of cortical rhythms in mild Alzheimer's disease. A multicentric EEG study. *NeuroImage*.

[3] Babiloni C, Frisoni G, Steriade M (2006). Frontal white matter volume and delta EEG sources negatively correlate in awake subjects with mild cognitive impairment and Alzheimer's disease. *Clinical Neurophysiology*.

[4] Babiloni C, Benussi L, Binetti G (2006). Apolipoprotein E and alpha brain rhythms in mild cognitive impairment: a multicentric electroencephalogram study. *Annals of Neurology*.

[5] Tsuno N, Shigeta M, Hyokid K, Faber PL, Lehmann D (2004). Fluctuations of source locations of EEG activity during transition from alertness to sleep in Alzheimer's disease and vascular dementia. *Neuropsychobiology*.

[6] Huang C, Wahlund L-O, Dierks T, Julin P, Winblad B, Jelic V (2000). Discrimination of Alzheimer's disease and mild cognitive impairment by equivalent EEG sources: a cross-sectional and longitudinal study. *Clinical Neurophysiology*.

[7] Dierks T, Ihl R, Frölich L, Maurer K (1993). Dementia of the Alzheimer type: effects on the spontaneous EEG described by dipole sources. *Psychiatry Research*.

[8] Dierks T, Jelic V, Pascual-Marqui RD (2000). Spatial pattern of cerebral glucose metabolism (PET) correlates with localization of intracerebral EEG-generators in Alzheimer's disease. *Clinical Neurophysiology*.

[9] Dierks T, Frölich L, Ihl R, Maurer K (1995). Correlation between cognitive brain function and electrical brain activity in dementia of Alzheimer type. *Journal of Neural Transmission*.

[10] Hara J, Shankle WR, Musha T (1999). Cortical atrophy in Alzheimer's disease unmasks electrically silent sulci and lowers EEG dipolarity. *IEEE Transactions on Biomedical Engineering*.

[11] Huang C, Wahlund L-O, Dierks T, Julin P, Winblad B, Jelic V (2000). Discrimination of Alzheimer's disease and mild cognitive impairment by equivalent EEG sources: a cross-sectional and longitudinal study. *Clinical Neurophysiology*.

[12] Bennys K, Rondouin G, Vergnes C, Touchon J (2001). Diagnostic value of quantitative EEG in Alzheimer's disease. *Neurophysiologie Clinique*.

[13] Nuwer M (1997). Assessment of digital EEG, quantitative EEG, and EEG brain mapping: report of the American Academy of Neurology and the American Clinical Neurophysiology Society. *Neurology*.

[14] Adler G, Brassen S, Jajcevic A (2003). EEG coherence in Alzheimer's dementia. *Journal of Neural Transmission*.

[15] Musha T, Asada T, Yamashita F (2002). A new EEG method for estimating cortical neuronal impairment that is sensitive to early stage Alzheimer's disease. *Clinical Neurophysiology*.

[16] Melissant C, Ypma A, Frietman EEE, Stam CJ (2005). A method for detection of Alzheimer's disease using ICA-enhanced EEG measurements. *Artificial Intelligence in Medicine*.

[17] Petrosian AA, Prokhorov DV, Lajara-Nanson W, Schiffer RB (2001). Recurrent neural network-based approach for early recognition of Alzheimer's disease in EEG. *Clinical Neurophysiology*.

[18] Vialatte F, Cichocki A, Dreyfus G, Musha T, Shishkin SL, Gervais R Early detection of Alzheimer's disease by blind source separation, time frequency representation, and bump modeling of EEG signals.

[19] Jeong J (2004). EEG dynamics in patients with Alzheimer's disease. *Clinical Neurophysiology*.

[20] Cichocki A, Shishkin SL, Musha T, Leonowicz Z, Asada T, Kurachi T (2005). EEG filtering based on blind source separation (BSS) for early detection of Alzheimer's disease. *Clinical Neurophysiology*.

[21] Cichocki A (2004). Blind signal processing methods for analyzing multichannel brain signals. *International Journal of Bioelectromagtism*.

[22] Cichocki A, Amari S-I (2003). *Adaptive Blind Signal and Image Processing: Learning Algorithms and Applications*.

[23] Rumelhart DE, Smolensky P, McClelland JL, Hinton GE, McClelland JL, Rumelhart DE (1986). Schemata and sequential thought processes in PDP models. *Parallel Distributed Processing: Explorations in the Microstructure of Cognition*.

[24] Buscema M (1998). Constraint satisfaction neural networks. *Substance Use & Misuse*.

[25] Buscema M (1998). Recirculation neural networks. *Substance Use & Misuse*.

[26] Hinton GE, McClelland JL Learning representation by recirculation.

[27] Chauvin Y, Rumelhart DE (1995). *Backpropagation: Theory, Architectures, and Applications*.

[28] Elman JL (1990). Finding structure in time. *Cognitive Science*.

[29] Buscema M I FAST Software, Semeion Software #32.

[30] Buscema M, Grossi E, Intraligi M, Garbagna N, Andriulli A, Breda M (2005). An optimized experimental protocol based on neuro-evolutionary algorithms: application to the classification of dyspeptic patients and to the prediction of the effectiveness of their treatment. *Artificial Intelligence in Medicine*.

[31] Buscema M TWIST Software, Semeion Software #32.

[32] Buscema M (2004). Genetic doping algorithm (GenD): theory and applications. *Expert Systems*.

[33] Davis L (1991). *Handbook of Genetic Algorithms*.

[34] Harp S, Samed T, Guha A, Touretzky D (1990). Designing application-specific neural networks using the genetic algorithm. *Advances in Neural Information Processing Systems*.

[35] Mitchell M (1996). *An Introduction to Genetic Algorithms*.

[36] Quagliarella D, Periaux J, Polani C, Winter G (1998). *Genetic Algorithms and Evolution Strategies in Engineering and Computer Science*.

[37] Rawling G (1991). *Foundations of Genetic Algorithms*.

[38] Dietterich TG (1998). Approximate statistical tests for comparing supervised classification learning algorithms. *Neural Computation*.

[39] Rubin EH, Morris JC, Grant FA, Vendegna T (1989). Very mild senile dementia of the Alzheimer type I. Clinical assessment. *Archives of Neurology*.

[40] Albert M, Smith LA, Scherr PA, Taylor JO, Evans DA, Funkenstein HH (1991). Use of brief cognitive tests to identify individuals in the community with clinically diagnosed Alzheimer’s disease. *International Journal of Neuroscience*.

[41] Flicker C, Ferris SH, Reisberg B (1991). Mild cognitive impairment in the elderly. *Neurology*.

[42] Zaudig M (1992). A new systematic method of measurement and diagnosis of “mild cognitive impairment” and dementia according to ICD-10 and DSM-III-R criteria. *International Psychogeriatrics*.

[43] Devanand DP, Folz M, Gorlyn M, Moeller JR, Stern Y (1997). Questionable dementia: clinical course and predictors of outcome. *Journal of the American Geriatrics Society*.

[44] Petersen RC, Smith GE, Ivnik RJ (1995). Apolipoprotein E status as a predictor of the development of Alzheimer’s disease in memory-impaired individuals. *Journal of the American Medical Association*.

[45] Petersen RC, Smith GE, Waring SC, Ivnik RJ, Kokmen E, Tangelos EG (1997). Aging, memory, and mild cognitive impairment. *International Psychogeriatrics*.

[46] Petersen RC, Doody R, Kurz A (2001). Current concepts in mild cognitive impairment. *Archives of Neurology*.

[47] McKhann G, Drachman D, Folstein M, Katzman R, Price D, Stadlan EM (1984). Clinical diagnosis of Alzheimer’s disease: report of the NINCDS-ADRDA work group under
the auspices of department of health and human services task force on Alzheimer’s disease. *Neurology*.

[48] Folstein MF, Folstein SE, McHugh PR (1975). Mini mental state: a practical method for grading the cognitive state of patients for the clinician. *Journal of Psychiatric Research*.

[49] Hughes CP, Berg L, Danziger WL, Coben LA, Martin RL (1982). A new clinical scale for the staging of dementia. *The British Journal of Psychiatry *.

[50] Yesavage JA, Brink TL, Rose TL (1983). Development and validation of a geriatric depression screening scale: a preliminary report. *Journal of Psychiatric Research*.

[51] Rosen WG, Terry RD, Fuld PA, Katzman R, Peck A (1980). Pathological verification of ischemic score in differentiation of dementias. *Annals of Neurology*.

[52] Lawton MP, Brody EM (1969). Assessment of older people: self maintaining ad instrumental activities of daily living. *Gerontologist*.

[53] Brun A, Englund B, Gustafson L (1994). Consensus on clinical and neuropathological criteria for fronto-temporal dementia. * Journal of Neurology, Neurosurgery and Psychiatry*.

[54] Roman GC, Tatemichi TK, Erkinjuntti T (1993). Vascular dementia: diagnostic criteria for research studies: report of the NINDS-AIREN international workshop. *Neurology*.

[55] Frisoni GB, Beltramello A, Binetti G (1995). Computed tomography in the detection of the vascular component in dementia. *Gerontology*.

[56] Galluzzi S, Sheu CF, Zanetti O, Frisoni GB (2005). Distinctive clinical features of mild cognitive impairment with subcortical cerebrovascular disease. *Dementia and Geriatric Cognitive Disorders*.

[57] McKeith IG, Galasko D, Kosaka K (1996). Consensus guidelines for the clinical and pathologic diagnosis of dementia with Lewy
bodies (DLB): report of the consortium on DLB international workshop. *Neurology*.

[58] Buchan RJ, Nagata K, Yokoyama E (1997). Regional correlations between the EEG and oxygen metabolism in dementia of Alzheimer’s type. *Electroencephalography and Clinical Neurophysiology*.

[59] Pucci E, Belardinelli N, Cacchiò G, Signorino M, Angeleri F (1999). EEG power spectrum differences in early and late onset forms of Alzheimer’s disease. *Clinical Neurophysiology*.

[60] Szelies B, Mielke R, Kessler J, Heiss W-D (1999). EEG power changes are related to regional cerebral glucose metabolism in vascular dementia. *Clinical Neurophysiology*.

[61] Rodriguez G, Vitali P, De Leo C, De Carli F, Girtler N, Nobili F (2002). Quantitative EEG changes in Alzheimer patients during long-term donepezil therapy. *Neuropsychobiology*.

[62] Babiloni C, Ferri R, Moretti DV (2004). Abnormal fronto-parietal coupling of brain rhythms in mild Alzheimer’s disease: a multicentric EEG study. *European Journal of Neuroscience*.

[63] Moretti DV, Babiloni F, Carducci F (2003). Computerized processing of EEG-EOG-EMG artifacts for multi-centric studies in
EEG oscillations and event-related potentials. *International Journal of Psychophysiology*.

[64] Stern Y (2006). Cognitive reserve and Alzheimer disease. *Alzheimer Disease and Associated Disorders*.

[65] Gauthier SG (2005). Alzheimer’s disease: the benefits of early treatment. *European Journal of Neurology*.

